# Genetic lineages of undifferentiated-type gastric carcinomas analysed by unsupervised clustering of genomic DNA microarray data

**DOI:** 10.1186/1755-8794-6-25

**Published:** 2013-07-19

**Authors:** Ayano Sonoda, Ken-ichi Mukaisho, Takahisa Nakayama, Vo Thi Ngoc Diem, Takanori Hattori, Akira Andoh, Yoshihide Fujiyama, Hiroyuki Sugihara

**Affiliations:** 1 Department of Pathology, Division of Molecular and Diagnostic Pathology, Shiga University of Medical Science, Otsu 520-2192, Japan; 2 Department of Internal Medicine, Division of Gastroenterology and Hematology, Shiga University of Medical Science, Otsu 520-2192, Japan

## Abstract

**Background:**

It is suspected that early gastric carcinoma (GC) is a dormant variant that rarely progresses to advanced GC. We demonstrated that the dormant and aggressive variants of tubular adenocarcinomas (TUBs) of the stomach are characterized by loss of *MYC* and gain of *TP53* and gain of *MYC* and/or loss of *TP53*, respectively. The aim of this study is to determine whether this is also the case in undifferentiated-type GCs (UGCs) of different genetic lineages: one with a layered structure (LS+), derived from early signet ring cell carcinomas (SIGs), and the other, mostly poorly differentiated adenocarcinomas, without LS but with a minor tubular component (TC), dedifferentiated from TUBs (LS−/TC+).

**Methods:**

Using 29 surgically resected stomachs with 9 intramucosal and 20 invasive UGCs (11 LS+ and 9 LS−/TC+), 63 genomic DNA samples of mucosal and invasive parts and corresponding reference DNAs were prepared from formalin-fixed, paraffin-embedded tissues with laser microdissection, and were subjected to array-based comparative genomic hybridization (aCGH), using 60K microarrays, and subsequent unsupervised, hierarchical clustering. Of 979 cancer-related genes assessed, we selected genes with mean copy numbers significantly different between the two major clusters.

**Results:**

Based on similarity in genomic copy-number profile, the 63 samples were classified into two major clusters. Clusters A and B, which were rich in LS+ UGC and LS−/TC+ UGC, respectively, were discriminated on the basis of 40 genes. The aggressive pattern was more frequently detected in LS−/TC+ UGCs, (20/26; 77%), than in LS+UGCs (17/37; 46%; P = 0.0195), whereas no dormant pattern was detected in any of the UGC samples.

**Conclusions:**

In contrast to TUBs, copy number alterations of *MYC* and *TP53* exhibited an aggressive pattern in LS+ SIG at early and advanced stages, indicating that early LS+ UGCs inevitably progress to an advanced GC. Cluster B (enriched in LS−/TC+) exhibited more frequent gain of driver genes and a more frequent aggressive pattern than cluster A, suggesting potentially worse prognosis in UGCs of cluster B.

## Background

Gastric carcinoma (GC) have been classified histologically into intestinal, diffuse and unclassified types by Lauren [1] and the unclassified type was further divided into solid and mixed types by Carneiro [2]. The undifferentiated-type gastric carcinoma (UGC) according to the Japanese classification [3] mostly overlaps poorly differentiated GC, which comprises not only the diffuse type including signet ring cell carcinoma (SIG) but also the solid type and the mixed type with minor tubular component (TC).

Recently it has been proposed that advanced diffuse-type GC may derive from either early diffuse-type or intestinal-type GC. Well differentiated tubular adenocarcinoma (TUB) can transform into poorly differentiated adenocarcinoma (POR) after the silencing of cell adhesion-related genes including *CDH1*[4,5]. Carneiro’s mixed type carcinomas may thus overlap dedifferentiated TUBs. It has been reported that the survival rate of the patients with mixed-type GCs was significantly lower than that of the patients with GCs of other types [2], whereas the survival rate of early GC patients with SIG was higher than that of GC patients without SIG [6]. Thus UGCs may be divided into subgroups with different prognosis. Recently, a mass-screening program for neuroblastomas [7-9] was suspended in Japan because a discontinuous genetic lineage was observed between the early- and the late-presenting neuroblastomas. Negative and late-presenting (≥1 year) neuroblastomas exhibited near-diploidy with terminal 1p deletion, whereas positive neuroblastomas in infants exhibited near-triploidy without 1p deletion [10,11]. To perform such subgrouping, we have classified UGCs based on the continuity of genetic lineages as well as the expression of morphological lineage markers.

Our lineage analysis using chromosomal comparative genomic hybridization (CGH) was based on distinctive morphological lineage markers. A layered structure (LS) represents an incipient phase of SIG development [12] and is commonly retained even at an advanced stage in the human stomach. In tumour regions with LS, the mode of cell proliferation resembles that in the normal gastric mucosa. And it is believed that tumour cells remain confined to the mucosa as far as they grow to form the LS [13]. Our lineage analyses confirmed that POR with LS was derived from intramucosal SIG, whereas POR without LS and with a minor TC (< 30%), was derived from TC [14,15]. However, the TC was not always derived from early TUB but could also be derived from SIG, whereas LS was scarcely derived from TUB [15]. Therefore, as a morphological lineage marker, LS may take priority over TC. In addition, UGCs without LS or TC due to secondary loss of these markers are observed, which prompted us to adopt array CGH (aCGH) and unsupervised cluster analyses of the aCGH data to classify UGCs solely on the basis of similarity in the genomic copy number profile.

In differentiated-type gastric carcinomas (DGCs), our recent aCGH-based lineage analyses revealed two genetic lineages: one with copy-number loss of *MYC* and copy-number gain of *TP53* (*MYC*− and *TP53+*), a dormant pattern, and the other with the copy-number gain of *MYC* and/or copy-number loss of *TP53* (*MYC+* and/or *TP53*−), an aggressive pattern. The dormant pattern accounted for 70% of intramucosal carcinoma samples and a half of the intramucosal part samples of invasive carcinomas. The invasive parts of invasive carcinomas mostly exhibited the aggressive copy number alteration (CNA) pattern. When the intramucosal part of an advanced cancer was dormant, the lineage was discontinuous between the mucosal and invasive parts. Therefore, the *MYC*−/*TP53*+ and *MYC*+ and/or *TP53*− CNA patterns may be signatures of dormant and aggressive TUBs, respectively [16].

In the present study, genomic DNA samples from the mucosal and invasive parts of early and advanced UGCs were prepared and subjected to gene copy-number analyses using aCGH, followed by unsupervised cluster analysis of the aCGH data. Based on these results, we examined relationship between morphological and genetic lineage markers and identified several useful lineage marker genes for UGC.

## Methods

The Institutional Review Board on Medical Ethics at Shiga University of Medical Science approved this study on the condition that the UGC samples used were anonymous. Written informed consent was not required because this retrospective study used archival samples.

### Tissue samples

This study included 29 surgically resected, buffered formalin-fixed, paraffin embedded UGCs: 20 with LS in at least part of the tumour (LS+, 9 intramucosaltumours and 11 invasive tumours) and 9 without LS but containing a small TC (LS−/TC+, all invasive tumours) (Table 1). TC was defined as a well or moderately differentiated adenocarcinoma component comprising ≤ 30% of the entire tumour [15]. All samples were selected from GC cases diagnosed in our department from 1997 to 2011. Intramucosal LS+ UGC patients averaged 57.6 years of age (range, 48–79) and patients with invasive LS+ UGCs 60.2 years (range, 48–79) and patients with invasive LS−/TC+ UGCs 62.2 years (range; 50–75). The macroscopic classification was determined according to the Japanese Classification of Gastric Cancer with TNM staging [3].

**Table 1:** Summary of clinicopathological characteristics of 29 UGCs

**Case no**	**Age/sex**	**Size of mucosal lesion (cm)**	Macroscopic type*	Histological type*	**Sampling region for aCGH**				**Depth of invasion†**	**LN meta†**	**Stage†**
					**Intramucosal part**			**Invasive part**			
					**LS**	**not LS**	**TC**				
M101	79/F	8.5 × 4.0	0(IIc)	SIG > TC	+	NT	NT		T1 (m)	N0	IA
M102	48/F	9.5 × 5.0	0(IIc)	SIG > POR1	+	NT	-		T1 (m)	N0	IA
M103	57/M	1.4 × 0.8	0(IIc)	SIG	+	NT	-		T1 (m)	N0	IA
M104	76/F	6.0 × 5.0	0(IIc)	SIG	+	NT	-		T1 (m)	N0	IA
M105	50/M	1.5 × 1.2	0(IIc)	SIG	+	NT	-		T1 (m)	N0	IA
M106	60/F	1.2 × 1.0	0(IIc)	SIG	+	-	-		T1 (m)	N0	IA
M107	49/F	4.0 × 2.5	0(IIc + III)	SIG > POR1	+	NT	-		T1 (m)	N0	IA
M108	48/F	6.0 × 2.8	0(IIc)	SIG > POR1 > TC	+	POR	NT		T1 (m)	N0	IA
M109	51/M	5.3 × 3.3	0(IIc + III)	SIG	+	SIG	-		T1 (m)	N0	IA
SM101	71/F	0.9 × 0.8	0(IIc)	SIG > POR2	+	NT	-	NT	T1 (sm2)	N2	II
A102	72/F	5.0 × 3.0	0(IIc + IIb)	POR2 > POR1 > SIG	+	SIG	-	POR2	T2 (mp)	N1	II
A103	79/F	12.0 × 8.5	0(IIa + IIb)	POR1 > TC > SIG > POR2	+	POR	NI	NT	T2 (mp)	N1	II
A104	49/M	2.8 × 2.5	0(IIc + III)	SIG > POR2 > TC	+	NT	NT	POR2	T2 (ss)	N1	II
SM105	59/F	11.5 × 7.0	0(IIa + IIc)	SIG > TC > POR2	+	TUB2	NT	POR2	T1 (sm)	N1	IB
SM106	72/M	3.7 × 2.3	0(IIc)	SIG > POR1	+	POR	-	NT	T1 (sm2)	N0	IA
A107	48/F	12.0 × 6.5	0(IIc + III)	SIG > POR2 > POR1 > MUC	+	POR	-	POR2	T3 (se)	N2	IIIB
A108	46/M	4.0 × 2.8	0(IIc + III)	POR2 > POR1 > SIG	NI	NI	-	POR2	T2 (mp)	N2	IIIA
A109	55/M	3.8 × 3.3	0(IIc + III)	SIG > POR1	+	POR	-	SIG	T1 (sm2)	N0	IA
A110	57/M	5.5 × 2.2	0(IIc)	POR2 > SIG > TC	+	POR	-	POR2	T2 (mp)	N1	II
A111	54/F	8.0 × 7.0	3	POR2 > SIG	+	POR	-	POR2	T3 (se)	N2	IIIB
SM201	75/F	3.7 × 3.0	2	POR1 > TC	-	POR/TUB2	+	POR	T1 (sm2)	N0	IA
A202	60/M	4.0 × 3.8	0(IIc)	POR2 > POR1 > TC > SIG	-	POR/TUB2	+	POR	T2 (mp)	N0	IB
SM203	71/M	4.5 × 2.0	0(IIa + IIc)	POR1 > TC > SIG	-	POR/TUB2	+	POR	T1 (sm2)	N0	IA
A204	65/F	5.5 × 3.0	3	POR2 > TC > POR1	-	POR/TUB2	+	POR	T3 (se)	N3	IV
A205	54/M	7.4 × 5.8	5	POR2 > TC > POR1	-	POR/TUB2	+	POR	T3 (se)	N0	II
A206	67/F	5.5 × 4.0	4	POR2 > TC > POR1	-	POR/TUB2	+	NT	T4 (si)	N2	IV
A207	52/M	6.0 × 4.0	4	POR1 > POR2 > SIG > TC	-	POR/TUB2	+	POR	T3 (se)	N3	IV
A208	75/M	9.0 × 7.0	2	POR1 > TC	-	POR/TUB2	+	POR	T2 (mp)	N1	II
A209	50/F	2.3 × 0.8	3	POR2 > SIG > POR1 > TC	-	POR/TUB2	+	POR	T2 (mp)	N2	IIIA

*Japanese classification of gastric carcinoma, modified.

†TNM classification.

*UGCs*, Undifferentiated gastric carcinomas; *aCGH*, Array CGH; *LN*, Lymph node, *LS*, Layered structure; *TC*, Tubular component; *SIG*, Signet ring cell carcinoma; *POR*, Poorly differentiated adenocarcinoma; *POR1*, Solid POR; *POR2*, Non-solid POR; *TUB2*, Moderately differentiated adenocarcinoma; *MUC*, Mucinous adenocarcinoma; *m*, mucosa; *sm*, submucosa; *mp*, muscular propria; *ss*, subserora; *se*, serosal exposure; *si*, invasion to adjacent structures; *M*, Male; *F*, Female; *NT*, Not tested; *NI*, Not informative.

### LS evaluation

**Figure 1:**
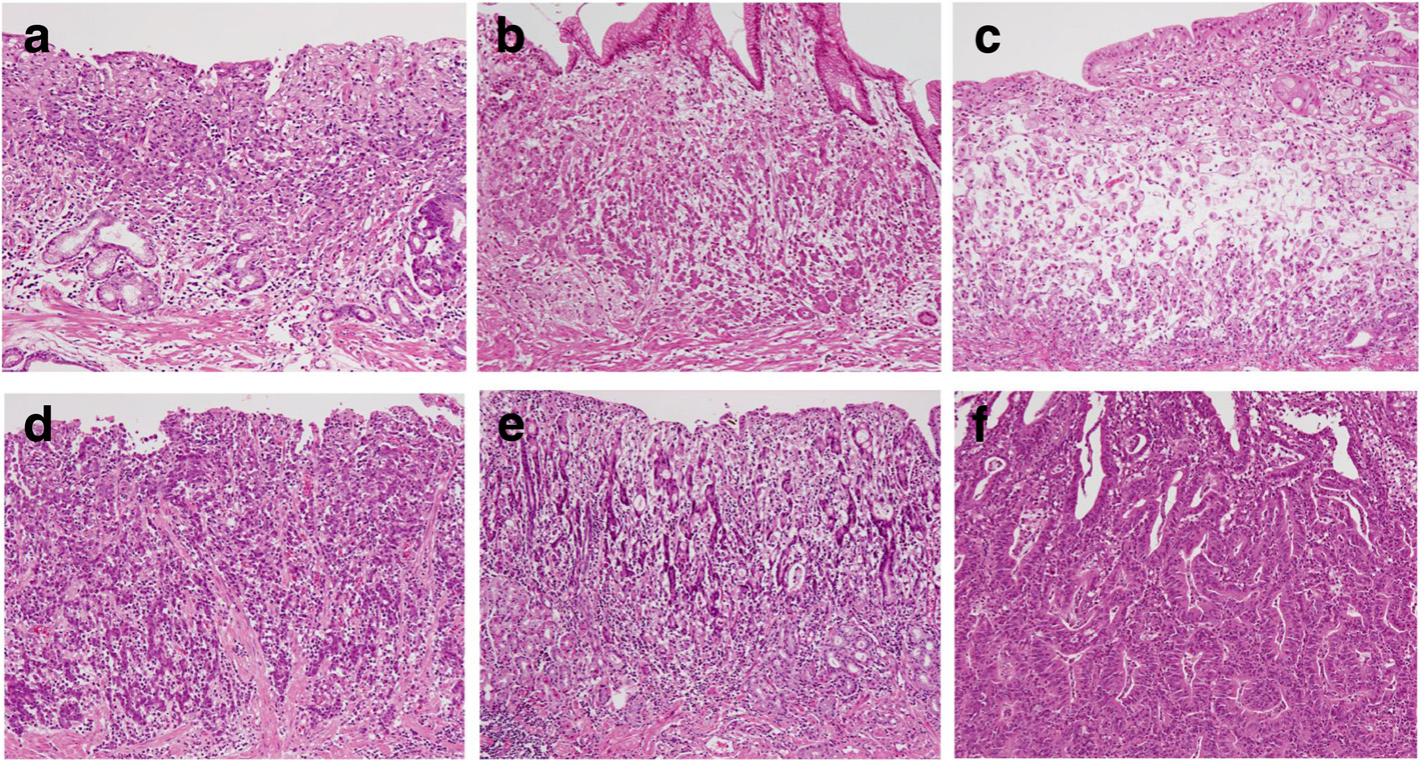
**Histological appearances of intramucosal parts of undifferentiated-type gastric carcinomas (UGCs).** A signet ring cell carcinoma (SIG) component with a layered structure in case A107 **(a)**. Small carcinoma cells are distributed in the deeper part just above or in the muscularis mucosae in a SIG component in case M109 **(b)**. A mucinous adenocarcinoma component in case A107 **(c)**. A poorly differentiated adenocarcinoma component in case SM106 **(d)**. Minor tubular components in cases SM105 and SM201, respectively **(e**, **f)**.

LS was defined as in a previous study [17]. In brief, LS*+* regions had small carcinoma cells confined to the stroma at the gland-neck level that gradually differentiated to signet ring cells in the superficial (and deep) lamina propria (Figure 1a). The absence of LS in intramucosal regions of the tumour was defined by four patterns: 1) contact of small carcinoma cells to the muscularis mucosae in SIG, 2) mucinous adenocarcinoma, 3) POR and 4) the presence of a TC (Figure 1b-f).

### Laser microdissection and DNA preparation

Tumour tissue samples were obtained from 5-μm-thick tissue sections using a LMD6000 laser microdissection system (Leica Microsystems, Wetzlar, Germany). For invasive cancers, DNA samples were obtained from both the intramucosal and invasive parts. For each sample, cancer tissues were obtained from an area >6 mm^2^, in which cancer cells accounted for ≥70% of the total cell count. Tissue samples were digested in 200 μg/ml proteinase K solution for approximately 72 hours at 37.0°C and genomic DNA extracted with phenol/chloroform.

### Whole genome amplification

Sample DNA was amplified using the GenomePlex Whole Genome Amplification Kit (WGA2 Kit; Sigma, St. Louis, USA) [18]. For some DNA samples that could not be sufficiently amplified, the WGA5 Kit (Sigma) was employed.

### Array CGH

An oligo CGH microarray (60K, 60-mer) (Agilent, Santa Clara, USA) was used in this study, according to the manufacturer's instructions. In brief, the amplified tumour and control DNA samples were non-enzymatically labelled with Cy5 and Cy3, respectively, using the Genome DNA ULS Labelling Kit (Agilent) and competitively hybridized to the microarray. The hybridized array images were captured using a DNA microarray scanner (Agilent) and then the fluorescence intensity of the tumour and control at each probe dot was calculated by Feature Extraction Ver.9.5.3 (Agilent). The array data were normalized using Genomic Workbench software Ver.5.0 (Agilent). The positions of oligomers are based on the Human Genome February 2009 assembly (hg19). Copy-number gains and losses were defined as changes in the logarithm to the base 2 of the tumour to reference signal intensity ratio (T/R) greater than 0.3219 and less than −0.3219, respectively.

### Cluster analysis

To perform novel subtyping of UGC samples based on genomic profile similarity in this study, an unsupervised hierarchical cluster analysis was applied across 63 samples from 29 UGC cases by using the Cluster 3.0 and TreeView software programs. The clustering algorithm was set to complete linkage clustering using an uncentered correlation. To enable unsupervised cluster analysis, we performed unbiased reduction in probe number from around 60,000 to several thousands of probes. For this purpose, we selected large genes because the greater number of corresponding probes resulted in improved signal-to-noise ratio of the representative gene copy numbers. The unsupervised strategy enabled us to set an internal standard to validate clustering results; the copy number profiles in samples of the same tumour should be more similar than any copy number profiles from another tumour because the gene alterations in the process of carcinogenesis are largely common among the samples from the same tumour.

### Statistical analyses

Differences in contingency tables were assessed for statistical significance using Fisher's exact test. A P < 0.05 (2-sided) was considered statistically significant. The Welch’s *t* test was used to evaluate the difference in mean DNA copy number for each probe between two clusters of samples. The Bonferroni correction was used to correct for multiple comparisons.

## Results

### Samples analysed with array CGH

Tissue samples were excised from 29 archived GC specimens by laser microdissection. The tissue sample population included 11 regions (from 9 intramucosal SIGs), of which 9 regions were LS+ and the other two LS−, 26 regions (from 11 LS+ invasive UGC), of which 10 were LS+ mucosal regions, 8 were LS− mucosal regions and 8 were invasive regions, and 26 regions (from 9 LS−/TC+ invasive UGCs): 9 intramucosal POR, 9 intramucosal TC and 8 invasive regions.

### Genome wide copy number alterations

**Figure 2:**
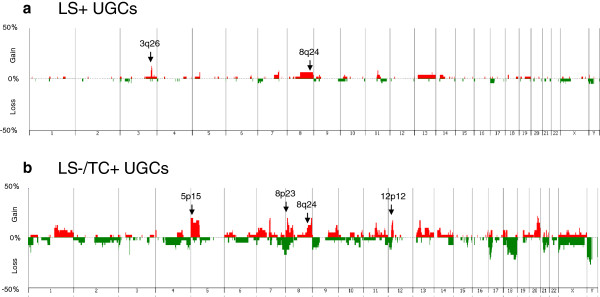
**Frequency of copy-number alterations at the chromosome level.** The percentage of the samples that have CNAs for each chromosome in the LS+ UGCs **(a)** and LS**−**/TC+ UGCs **(b)**. Gains and losses are indicated with red and green, respectively.

A plot of the genetic aberration penetrance for all chromosomes is shown for LS+ UGCs and LS−/TC+ UGCs in Figure 2a and Figure 2b, respectively. Copy-number gains and losses were more common in LS−/TC+ UGCs than in LS+ UGCs. The most frequent copy-number gains were detected at 3q26 (7/63 samples), 5p15 (8/63), 8p23 (9/63), 8q24 (7/63) and 12p12 (6/63), while the most frequent copy-number losses were found at 7q36 (5/63) and 12p12 (5/63).

Copy-number alterations (CNAs) common to all the samples from the same tumour were called stemline changes [14] and estimated to occur at the earliest stage of tumourigenesis and to be inherent into tumour lineage. Stemline gains of 3q26 were detected in 2/20 cases of invasive LS+ UGCs and none of invasive LS−/TC+ UGCs. In contrast, stemline gains of 5p15, 8p23 and 12p12 were detected in 2/9 cases of invasive LS−/TC+ UGCs but in no case of invasive LS+ UGCs. No stemline losses were detected in any cases of UGCs.

Previous studies using chromosomal or array CGH analyses [19-27] reported that frequent CNAs in gastric cancers (common to both UGC and DGC) were chromosomal gains at 3q, 5p, 7p, 8q, 13q, 17q, 20p and 20q, and losses at 4q, 5q, 6q, 9p, 17p, 18q and 21q. In the UGCs examined in the present study, all previously reported CNAs were observed except gains at 17q and 20p and losses at 5q and 6q. Gains at 8p and 12p were common in LS−/TC+ UGCs. Copy-number gains at 8q24 were common in both types of UGCs, with 4/20 cases of LS+ UGCs and 3/9 cases of invasive LS−/TC+ UGCs, but these were not stemline changes.

### Impartial selection of genes reflecting the whole genome profile

To classify UGC samples based on the overall similarity in the profile of gene copy number changes, we used unsupervised hierarchical cluster analysis. For this purpose, it was necessary to reduce the number of gene probes used in the cluster analysis from 60K to several thousands. The reduced number of genes should still reflect the whole genome profile if impartially selected. To fulfil these conditions, we selected genes based solely on the size of genes (the numbers of corresponding probes). After repeated trials of cluster analyses using genes of various minimum sizes (or probe numbers per gene), we observed that most CNAs from the same tumour were clustered more closely together than any samples from another UGC case when we analysed only genes with 3 or more probes per gene: a total of 5019 genes.

### Classification of UGC using hierarchical cluster analysis

**Figure 3:**
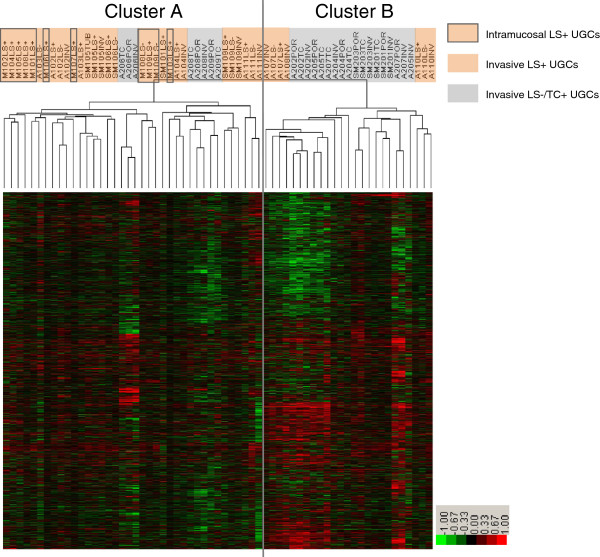
**Unsupervised hierarchical cluster analysis of array-based comparative genomic hybridization (aCGH) data.** Gene copy-number gains and losses are indicated by red and green, respectively. A total of 63 samples from 29 UGCs were classified into two major clusters: A and B. Most samples of LS+ UGCs were included in cluster A and most LS−/TC+ UGCs samples were in cluster B. All the Intramucosal LS+ UGCs were included in cluster A.

We applied an unsupervised two-dimensional hierarchical clustering algorithm, to a total of the 63 DNA samples from 29 UGCs. The samples were classified into two major clusters A and B, based on similarity in the genome profile (Figure 3). Of 63 samples, 30 LS+ UGCs were classified into cluster A and only 7 into cluster B. For LS−/TC+ UGCs, 8 samples were classified into cluster A and 18 into cluster B. All Intramucosal LS+ UGCs were included in cluster A. Clusters A and B had significantly different proportions of morphological subtypes (P = 0.0001).

### Copy number alterations of *MYC* and *TP53*

**Figure 4:**
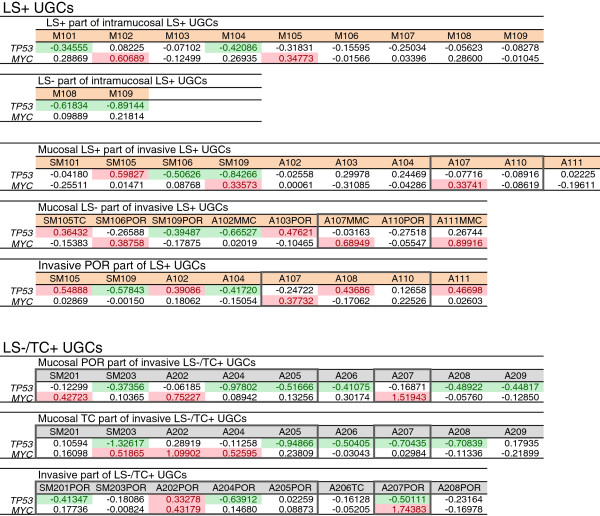
**Array CGH data of *MYC *and *TP53 *in LS+ UGCs and LS−/TC+ UGCs.** LS+ UGCs are divided into intramucosal cancers and invasive cancers. Numerals mean the base 2 logarithm of the test/reference signal intensity ratios of array CGH data. Significant gains and losses are indicated with red and green, respectively. The samples marked with and without grey margin are included in cluster B and cluster A, respectively, in Figure 3.

Gains at 8q24 were common alterations for both LS+ and LS−/TC+ UGCs. The representative genes located at this locus is *MYC.* Gains of *MYC* were detected in 2/11 of Intramucosal LS+ UGCs (18.2%), 6/26 of LS+ UGC (23.1%) and 8/26 of invasive LS−/TC+ UGCs (30.1%). The aggressive pattern (*MYC+* and/or *TP53*−) was detected in 6/11 of Intramucosal LS+ UGCs (54.5%), 11/26 of invasive LS+ UGCs (42.3%) and 20/26 of invasive LS−/TC+ UGCs (76.9%; Figure 4). Therefore, the aggressive pattern was more frequently detected in invasive LS−/TC+ UGCs than in LS+ UGCs (P = 0.0195). The dormant pattern (*MYC*− and *TP53+*) was not detected in any of the UGC samples, even those from intramucosal GCs (Figure 4).

### Copy number alterations of genes other than *MYC* or *TP53*

**Figure 5:**
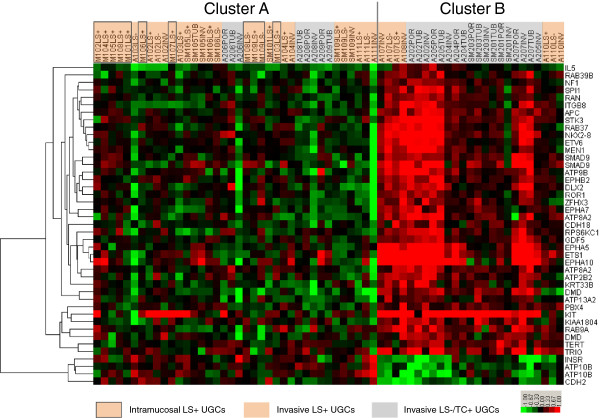
**Array CGH data of genes other than *MYC *and *TP53 *with significantly different T/R ratio between clusters A and B.** UGCs are divided into clusters A and B that were determined in Figure 3. The heat map indicates the base 2 logarithm of the test/reference signal intensity ratios of array CGH data. Gains and losses are indicated with red and green, respectively.

As mentioned above, 5p15 was one of the most frequent gain sites in invasive LS−/TC+ UGCs (8/26; 30.7%), but was not detected in any of the 37 intramucosal and invasive LS+ UGCs (Figure 2). The target genes located at this locus may include the telomerase reverse transcriptase gene (*TERT*) because a *TERT* gain was more frequently detected in invasive LS−/TC+ UGCs than intramucosal and invasive LS+ UGCs (16/26 vs. 1/37, P < 0.0001) (Figure 5). In contrast, losses of *TERT* were detected in 4/37 samples of intramucosal and invasive LS+ UGCs (10.8%) but not in invasive LS−/TC+ UGCs.

Welch’s *t* test was performed to compare the mean T/R ratio between the samples in cluster A and those in cluster B at each 2756 probe loci of 979 cancer-related genes. Fourty-three gene probes, belonging to 40 genes, had significantly different mean T/R ratios between Clusters A and B at a level of P < 0.05 after Bonferroni correction (Table 2). Of the 40 genes, 6 genes (*KIT*, *RAN*, *RAB39B*, *RAB9A*, *RAB37* and *TERT*), including proto-oncogenes, have been implicated in enhanced tumour growth, and 8 genes (*ETS1*, *SPI1*, *ETV6*, *EPHA7*, *EPHA5*, *EPHB2*, *EPHA10* and *TRIO*) in invasion/metastasis and 3 genes (*APC, NF1* and *MEN1*) in tumour suppression (Table 2). Most of log_2_ T/R ratios of the 43 distinguishing gene probes were of opposite sign between clusters A and B, with greater in absolute values in cluster B (Figure 5).

**Table 2:** List of 40 genes that have CNAs significantly different between clusters A and B

**Probe name**	**Location**	**Name of Gene**	**Description**	**P-value**	***P *value after Bonferroni correction**
A_16_P41637097	Xp21.2	*DMD*	dystrophin	4.541E-08	1.251E-04
A_14_P133591	5q21-q22	*APC*	adenomatous polyposis coli	5.774E-08	1.591E-04
A_14_P130973	4q11-q12	*KIT	v-kit Hardy-Zuckerman 4 feline sarcoma viral oncogene homolog	1.047E-07	2.886E-04
A_14_P125447	13q12-q14	*SMAD9*	SMAD family member 9	1.373E-07	3.784E-04
A_14_P100439	12q24.3	*RAN	member RAS oncogene family	1.876E-07	5.171E-04
A_14_P102616	20q11.2	*GDF5*	growth differentiation factor 5	2.828E-07	7.794E-04
A_14_P133647	11q23.3	**ETS1	v-etserythroblastosis virus E26 oncogene homolog 1 (avian)	5.603E-07	0.0015
A_14_P138640	5p14.3	*CDH18*	cadherin 18, type 2	6.138E-07	0.0017
A_14_P128664	18q23	*ATP9B*	ATPase, class II, type 9B	6.474E-07	0.0018
A_14_P124801	19p12	*PBX4*	pre-B-cell leukemia homeobox 4	1.010E-06	0.0028
A_14_P118423	Xq28	*RAB39B	member RAS oncogene family	1.573E-06	0.0043
A_14_P134602	17q11.2	*NF1*	neurofibromin 1	1.802E-06	0.0050
A_14_P120351	5q31.1	*IL5*	interleukin 5 (colony-stimulating factor, eosinophil)	1.988E-06	0.0055
A_14_P125637	6q16.1	**EPHA7	EPH receptor A7	2.153E-06	0.0059
A_14_P100300	13q12-q14	*SMAD9*	SMAD family member 9	2.525E-06	0.0070
A_14_P201681	7p21.1	*ITGB8*	integrin, beta 8	2.589E-06	0.0071
A_14_P130112	Xp22.2	*RAB9A	RAB9A, member RAS oncogene family	2.949E-06	0.0081
A_14_P120484	1q41	*RPS6KC1*	ribosomal protein S6 kinase, 52 kDa, polypeptide 1	3.052E-06	0.0084
A_16_P16709446	4q13.1	**EPHA5	EPH receptor A5	3.584E-06	0.0099
A_14_P201127	2q32	*DLX2*	distal-less homeobox 2	3.703E-06	0.0102
A_14_P126957	11p11.2	**SPI1	spleen focus forming virus (SFFV) proviral integration oncogene	4.334E-06	0.0119
A_14_P104667	8q22.2	*STK3*	serine/threonine kinase 3	4.386E-06	0.0121
A_14_P137889	14q13.3	*NKX2-8*	NK2 homeobox 8	5.393E-06	0.0149
A_14_P109970	1p36.1-p35	**EPHB2	Eph receptor B2	6.637E-06	0.0183
A_14_P118116	Xp21.2	*DMD*	dystrophin	8.087E-06	0.0223
A_16_P01378894	5q34	*ATP10B*	ATPase, class V, type 10B	8.165E-06	0.0225
A_14_P139456	17q25.1	*RAB37	member RAS oncogene family	1.001E-05	0.0276
A_14_P111361	17q21.2	*KRT33B*	keratin 33B	1.069E-05	0.0295
A_14_P134909	19p13.3-p13.2	*INSR*	insulin receptor	1.110E-05	0.0306
A_16_P02740008	13q12	*ATP8A2*	ATPase, aminophospholipid transporter, class I, type 8A, member 2	1.129E-05	0.0311
A_14_P102858	1q42	*KIAA1804*	mixed lineage kinase 4	1.155E-05	0.0318
A_14_P113857	12p13	**ETV6	ets variant 6	1.172E-05	0.0323
A_14_P115054	16q22.3	*ZFHX3*	zinc finger homeobox 3	1.180E-05	0.0325
A_14_P138431	1p32-p31	*ROR1*	receptor tyrosine kinase-like orphan receptor 1	1.209 E-05	0.0333
A_18_P22746653	3p25.3	*ATP2B2*	ATPase, Ca++ transporting, plasma membrane 2	1.282E-05	0.0353
A_14_P105811	11q13	*MEN1*	multiple endocrine neoplasia I	1.318E-05	0.0363
A_14_P136621	18q11.2	*CDH2*	cadherin 2, type 1, N-cadherin (neuronal)	1.420E-05	0.0391
A_14_P103176	1p34.3	**EPHA10	EPH receptor A10	1.455E-05	0.0401
A_16_P17370843	5q34	*ATP10B*	ATPase, class V, type 10B	1.556E-05	0.0429
A_14_P108129	5p15.33	*TERT	telomerase reverse transcriptase	1.584E-05	0.0436
A_16_P19750359	13q12	*ATP8A2*	ATPase, aminophospholipid transporter, class I, type 8A, member 2	1.586E-05	0.0437
A_14_P200005	1p36	*ATP13A2*	ATPase type 13A2	1.586E-05	0.0437
A_16_P01183532	5p15.2	**TRIO	trio Rho guanine nucleotide exchange factor	1.786E-05	0.0492

In the column of gene name, “*” indicates genes related to tumour growth and “**” those related to invasion and metastasis.

## Discussion

Based on chromosomal CGH analysis, we have reported that there are two distinct UGC lineages: the LS+ lineage derived from early SIG and LS−/TC+ lineage dedifferentiated from TUB [15]. The former is characterized by LS and the latter by a small TC. However, there are also UGCs without these morphological lineage markers. In the present study, we classified UGC based on similarity in the whole genome copy number profile among samples using unsupervised hierarchical cluster analysis and examined the correlation between this gene-based classification and morphological lineage markers.

Using 5019 large genes and aCGH data from 63 DNA samples from 29 UGCs, we confirmed that most of the samples examined from the same tumour were clustered more closely together than in any other sample, thus fulfilling the criteria for our internal standard. On the basis of this observation, we performed an unsupervised two-dimensional hierarchical cluster analysis. All the samples were classified into two major clusters A and B (Figure 3). Cluster A was rich in LS+ UGCs, whereas cluster B was rich in LS−/TC+ UGCs. This difference was statistically significant (P = 0.0001) and indicates that the classification by the presence or absence of LS and TC is well correlated with the genomic-profile-based classification and validates the LS+ and LS−/TC+ as lineage markers.

All the intramucosal LS+ UGCs were included in cluster A, suggesting that most of UGCs in cluster A were derived from intramucosal SIG, and that the LS−/TC+ UGCs in cluster A may have secondarily lost LS. The LS−/TC+ UGCs in cluster A may also be derived from SIG, as suggested by chromosomal CGH studies [15]. In contrast, LS in advanced LS+ UGCs in cluster B (A107, A108 and A110) was virtually indistinguishable morphologically but showed genomic constitutions different from LS in cluster A. This may be a kind of phenocopy; a fraction of LS+ UGCs were considerably similar in genomic profile to LS−/TC+ UGCs. Although LS exhibits regular cell proliferation and differentiation and a superficially spreading dormant growth [13], it is suggested that LS itself is not a marker of persistent tumour dormancy but has the potential to progress to an advanced stage with the prognosis as poor as that for LS−/TC+ UGCs. This situation may resemble that in chronic myeloid leukaemia, in which blastic transformation occurs after a dormant phase of well retained cellular differentiation.

Most UGCs exhibited the aggressive genomic pattern (*TP53*− and*/*or *MYC+*), even 55% of intramucosal LS+ UGCs, an incidence comparable to that in invasive UGCs. The dormant pattern (*MYC*− and *TP53*+) was not detected in any of the UGC samples, even in intramucosal UGCs. These intramucosal UGCs are distinct from early DGCs, in which 70% are of the dormant type [16]. Therefore, *TP53* and *MYC* are not as useful prognostic markers for UGCs.

To explore other genes important for differentiation of genetic lineage and for UGC prognosis, we first compared the profiles of chromosomal copy-number alterations (CNAs) between LS+ and LS−/TC+ UGCs. As shown in Figure 2, CNAs detected in LS+ tumours but not in LS−/TC+ tumours, include 3q26 gain, a locus likely to include *SKIL* because the average *SKIL* copy number was greater in LS+ tumours than in LS−/TC+ tumours (P = 0.0060). *SKIL* encodes SnoN protein that is proto-oncogenic by antagonizing cytostatic responses of TGF-β [28,29] and anti-oncogenic by activating p53 [30]. Those CNAs with the opposite pattern (present in LS−/TC+ tumours but not in LS+ tumours) were gains at 5p15, 8p23 and 12p12. The target genes at 5p15 and 12q12 include *TERT*, and *KRAS*, respectively because gains of *TERT*, and *KRAS* were more frequently detected in invasive LS−/TC+ UGCs than intramucosal and invasive LS+ UGCs (P < 0.0001 and P = 0.0032, respectively). No target gene was detected at 8q23.

Our second approach to identify lineage-specific CNAs was a screening of genes (from 979 cancer-related genes) that indicated significantly different mean T/R ratios between the samples of clusters A and B. We selected 40 genes that were significantly different between clusters (using *t* test after Bonferroni correction), of which 6 were related to enhanced tumour growth and 8 to invasion/metastasis (Table 2). As shown in Figure 5, genes that drive tumourigenesis were more common in cluster B and showed larger amplitude CNAs. Thus, UGCs in cluster B may be more dependent on oncogenic genomic alterations and less on environmental and epigenetic alterations than those in cluster A.

The possible drivers of tumour growth screened included *KIT, TERT*, and *RAS* family genes. *KIT* encodes a receptor tyrosine kinase that is activated by stem cell factor binding and initiates numerous signal transduction pathways linked with the process of apoptosis, proliferation and tumorigenesis [31]. *RAS* family genes encode small GTPase that plays a key role in transduction of signals from receptor kinase to the pathways of various cellular processes [32]. *TERT* encodes the telomerase catalytic subunit that plays not only an important role in cellular immortalization by telomere elongation [33,34] but also activates cell proliferation [35]. The possible drivers of invasion and metastasis screened include *ETS1* and Ephrin receptor genes. *ETS1* encodes a transcription factor, Ets1 proto-oncoprotein that promotes invasiveness and is an indicator of poor outcome in epithelial cancers through regulation of MMP1, MMP3, MMP9, uPA, VEGF and VEGF receptor expression [36]. Ephrin receptor genes, EPH39B, A7, A5 and A10 genes encode the ephrin receptor with tyrosine kinase activity that affects tumor growth, invasiveness, angiogenesis, and metastasis [37].

There were no significant differences in the mean copy number of *CDH1* and its transcriptional repressor genes (*SNAI1*, *SNAI2*, *ZEB1*, *ZEB2*, *TWIST1*, etc.) between the clusters A and B, although these genes were reportedly associated with a poorly differentiated phenotype and poor clinical outcome [38]. However, these genes may still participate in UGC tumourigenesis through epigenetic silencing [39].

We are now extending this study to to validate UGC-associated genes as indicated by aCGH by quantitative PCR and to correlate their genomic copy number to gene expression and prognosis. Thereafter, using quantitative PCR analyses instead of aCGH, similar analyses should be applied to a greater number of tumour cases with known outcomes.

## Conclusions

Unsupervised cluster analyses of aCGH data of multiple samples from early and advanced UGCs have demonstrated that early UGCs, including LS+ types in which polarity of cell proliferation and differentiation is well retained, have aggressive potential. Therefore, eradication of UGCs at early stages may thus contribute to better patient survival. In addition, it was observed that the two UGC lineages, one derived from early SIG and the other from TUB, have different genomic copy-number alteration profiles, resulting in different sets of genes contributing to tumourigenesis. The latter lineage from TUB may be more dependent on genomic copy-number alterations and have a poorer outcome than UGCs derived from SIG.

## Competing interests

The authors declare that they have no competing interests.

## Authors’ contributions

AS performed most of the experiments, participated in the experimental design performed most of the data analyses and drafted the manuscript. KM participated in sample preparation for array CGH. TN participated partly in cluster analysis. VTND participated in varying selections of genes for cluster analysis, and TH, AA and YF provided critical comments and suggested revisions to the manuscript. HS conceived the study, designed the research route, and guided the experiments and the data analyses. All authors have read and approved the final manuscript.

## Pre-publication history

The pre-publication history for this paper can be accessed here:

http://www.biomedcentral.com/1755-8794/6/25/prepub
